# Statistical Permutation-based Artery Mapping (SPAM): a novel approach to evaluate imaging signals in the vessel wall

**DOI:** 10.1186/s12880-017-0207-7

**Published:** 2017-05-26

**Authors:** Robert Seifert, Aaron Scherzinger, Friedemann Kiefer, Sven Hermann, Xiaoyi Jiang, Michael A. Schäfers

**Affiliations:** 1 European Institute for Molecular Imaging (EIMI), University of Münster, Münster, Germany; 2 Department of Mathematics and Computer Science, University of Münster, Münster, Germany; 3 Max Planck Institute for Molecular Biomedicine, Münster, Germany; 4 DFG Cluster of Excellence 1003 ’CiM - Cells in Motion’, Münster, Germany; 5 Department of Nuclear Medicine, University Hospital Münster, Münster, Germany

**Keywords:** Atherosclerosis, Molecular imaging, Permutation testing, Threshold free cluster enhancement (TFCE)

## Abstract

**Background:**

Cardiovascular diseases are the leading cause of death worldwide. A prominent cause of cardiovascular events is atherosclerosis, a chronic inflammation of the arterial wall that leads to the formation of so called atherosclerotic plaques. There is a strong clinical need to develop new, non-invasive vascular imaging techniques in order to identify high-risk plaques, which might escape detection using conventional methods based on the assessment of the luminal narrowing. In this context, molecular imaging strategies based on fluorescent tracers and fluorescence reflectance imaging (FRI) seem well suited to assess molecular and cellular activity. However, such an analysis demands a precise and standardized analysis method, which is orientated on reproducible anatomical landmarks, ensuring to compare equivalent regions across different subjects.

**Methods:**

We propose a novel method, Statistical Permutation-based Artery Mapping (SPAM). Our approach is especially useful for the understanding of complex and heterogeneous regional processes during the course of atherosclerosis. Our method involves three steps, which are (I) standardisation with an additional intensity normalization, (II) permutation testing, and (III) cluster-enhancement. Although permutation testing and cluster enhancement are already well-established in functional magnetic resonance imaging, to the best of our knowledge these strategies have so far not been applied in cardiovascular molecular imaging.

**Results:**

We tested our method using FRI images of murine aortic vessels in order to find recurring patterns in atherosclerotic plaques across multiple subjects. We demonstrate that our pixel-wise and cluster-enhanced testing approach is feasible and useful to analyse tracer distributions in FRI data sets of aortic vessels.

**Conclusions:**

We expect our method to be a useful tool within the field of molecular imaging of atherosclerotic plaques since cluster-enhanced permutation testing is a powerful approach for finding significant differences of tracer distributions in inflamed atherosclerotic vessels.

## Background

Cardiovascular diseases are the leading cause of death worldwide [1] and most prominently originate from atherosclerosis, a chronic inflammation of the arterial vessel wall [2]. The complex pathophysiology of atherosclerosis includes the accumulation of lipids, inflammatory cells and fibrous tissue in the arterial wall, leading to its thickening. This results in the formation of so called atherosclerotic plaques, which may be asymptomatic, or, if plaques compromise the vessel lumen, leads to a hypoperfusion (ischemia) of organs downstream of the respective artery. Life-threatening clinical complications of atherosclerosis mainly occur when the atherosclerotic plaque becomes unstable (vulnerable) and ruptures, which immediately triggers clot formation resulting in the thrombotic occlusion of the affected artery. As a consequence of this event, the blood flow to an organ is suddenly terminated, resulting in organ hypoxia and loss of tissues as occurs in the major clinical scenarios myocardial infarction, stroke and peripheral vascular disease. Numerous inflammatory processes and other mechanisms that contribute to plaque vulnerability have been described [3]. There is a clinical need to develop new, non-invasive vascular imaging techniques in order to identify high-risk plaques, which might escape detection using conventional methods based on the assessment of the luminal narrowing of an artery secondary to a plaque in the arterial wall. Such an early and non-invasive diagnosis could have a major impact on individual treatment decisions as well as efficiency and hence the overall survival of the population at risk.

In this respect, molecular imaging strategies based on fluorescent or radioactive tracers combined with the respective imaging modalities such as fluorescence reflectance imaging (FRI), positron emission tomography (PET), and single photon emission tomography (SPECT) seem well suited to assess molecular and cellular activity. Various approaches of this kind targeting vascular inflammation have been described (reviewed in [4]). However, analysing tracer distribution in the arterial wall of a given vessel or the entire vascular tree in whole-body images remains a huge challenge. This is due to (a) the complex and variable anatomy of arteries across model systems, but also patients, (b) the thin vascular wall (partial volume), and (c) the spill-in of signals into the vascular wall from either the blood or tissues/organs surrounding the vessel, and various other factors. To address these issues, a precise and standardized analysis method is required which orientates itself on anatomical landmarks, ensuring to compare equivalent regions across different subjects [5].

Image standardization approaches are a common method for comparing functional images across different subjects, like in functional magnetic resonance imaging (fMRI) or PET data [6, 7]. This is due to the fact that Statistical Parametric Mapping (SPM) image analysis is widely used in neuroimaging research [6, 8]. Since Holmes et al. [9] discovered the advances of permutation-based statistical testing, there have been many achievements in non-parametric based image testing [10, 11]. Generally, permutation-based image testing performs better than parametric approaches [12], which explains the preference for permutation testing in neuroimaging and for electroencephalography (EEG) data. Additionally, such methods can be combined with cluster-enhancement of pixels [13, 14]. However, to the best of our best knowledge, so far these strategies have not been applied to cardiovascular molecular imaging. We therefore propose a novel method, Statistical Permutation-based Artery Mapping (SPAM). Our approach is especially useful in understanding the complex and heterogeneous regional processes in the course of atherosclerosis.

We chose 2D fluorescence reflectance imaging (FRI) data sets of tracer distribution in murine aortic vessels as an example for the application of permutation testing in combination with a cluster-enhancement approach to standardized images. We demonstrate that our pixel-wise and cluster-enhanced testing approach is feasible and useful for analysing MMP tracer distributions in FRI data sets of 13 aortic vessels.

## Methods

**Fig. 1:**
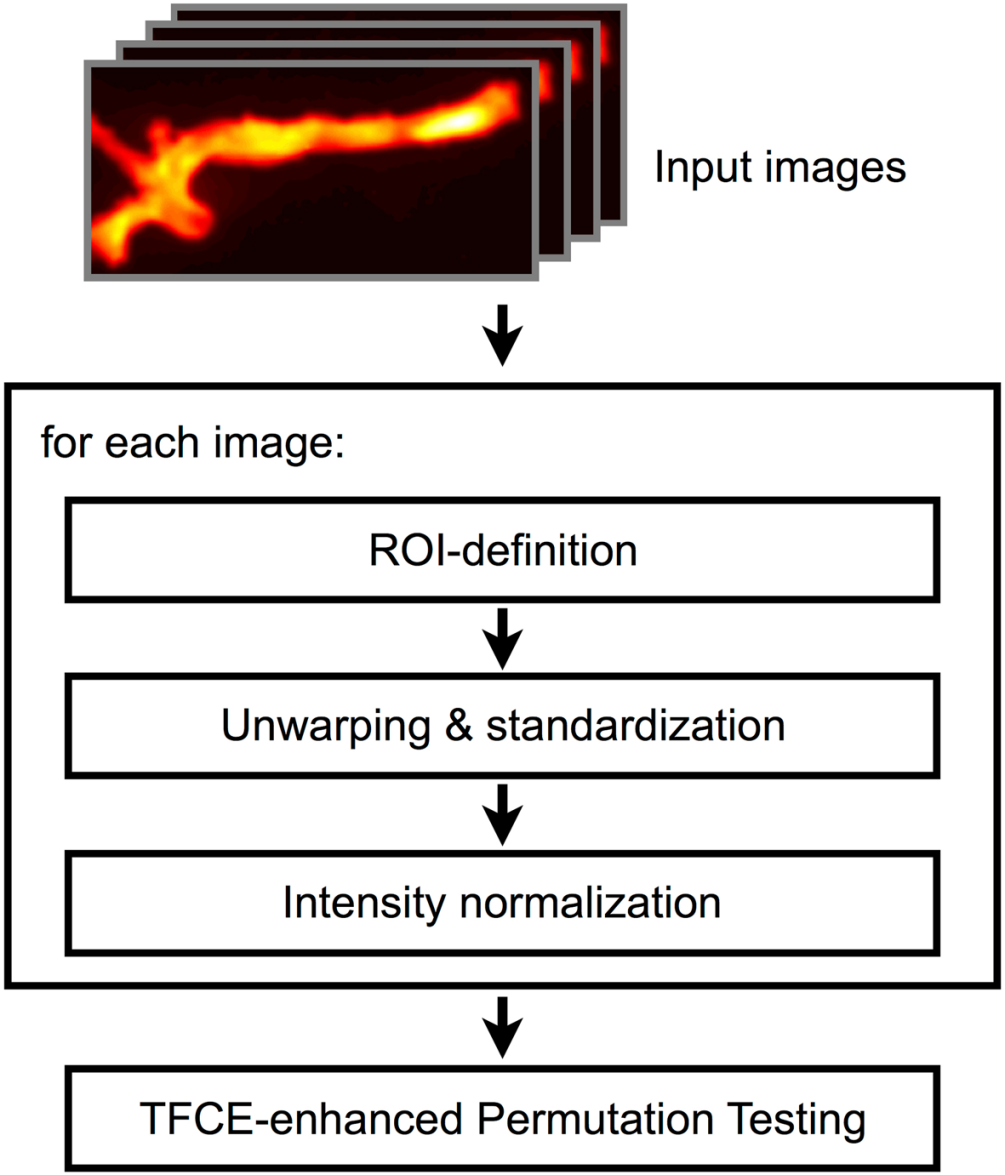
Overall Workflow, containing ROI definition, unwarping & standardization and intensity normalisation for every image of the test groups. Pre-processed map groups are then tested against each other using cluster enhanced permutations

**Fig. 2:**
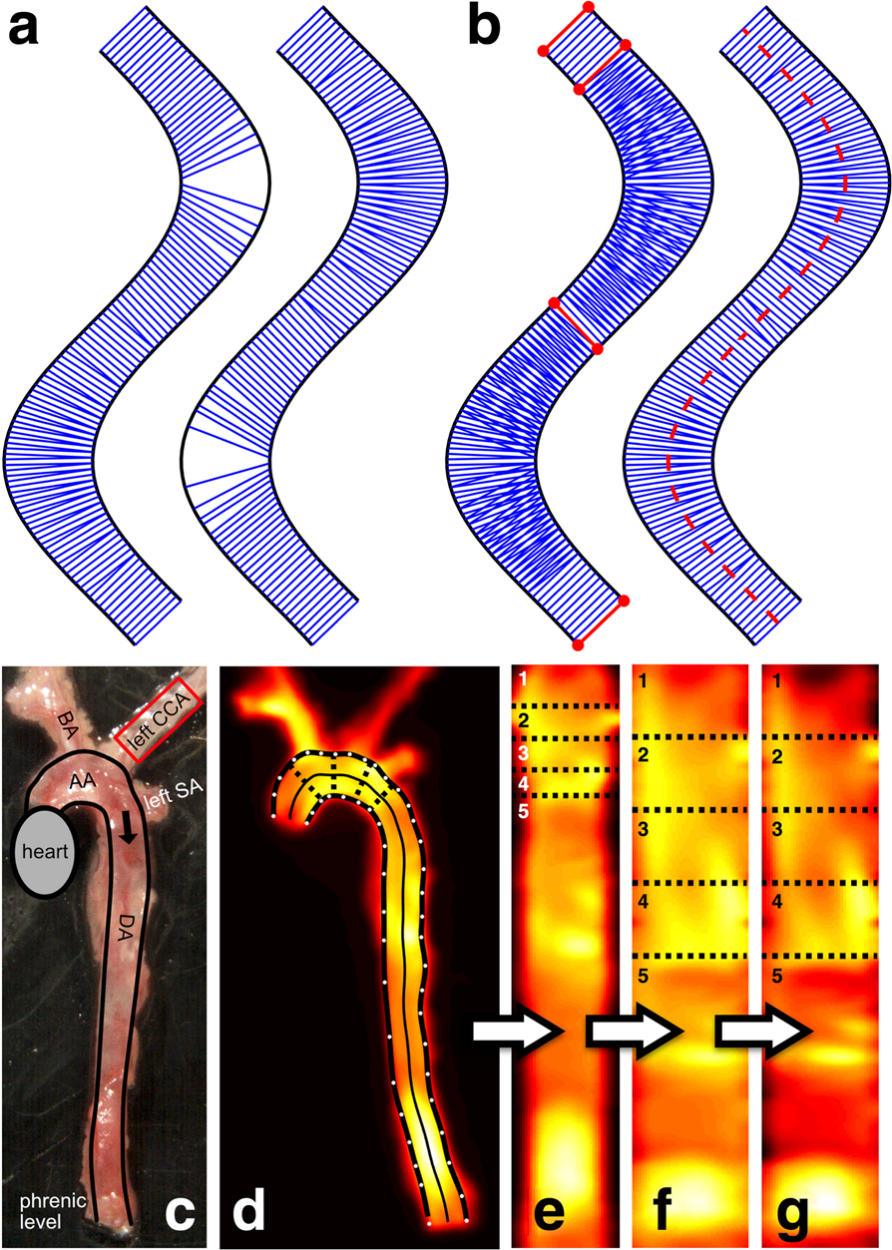
Schematic vessel boundaries marked as *black lines* in panel (**a**) and (**b**). Lines connecting nearest neighbours of points on the first boundary (panel **a** *left*) or the second boundary (panel **a** *right*) are drawn as *blue lines*. Common orthogonals between the two curves of the outer vessel boundaries are marked with *red lines* and points in panel (**b**). Choosing the boundary assignment for each segment between inflection points of the vessel separately leads to a set of non-crossing orthogonal line segments. In the right subfigure, the *red dashed line* shows the medial axis of the vessel computed from these non-crossing orthogonals. The microscopic photograph of the analysed aortic artery with highlighted boundaries is shown in panel (**c**). The ROI used for normalization is marked in *red*. (AA = aortic arch, DA = descending aorta, BA = brachycephalic artery, CCA = common carotid artery, SA = subclavian artery). Panel **d** shows the Cy5.5-AF443 fluorescence image where the manually defined boundary points are marked in *white*. Natural cubic spline curve interpolation of the boundary points leads to vessel boundaries marked in *bold black*. The *light black line* shows the medial axis of the vessel. The unwarped aortic vessel is shown in panel (**e**), numbers indicate corresponding vessel parts in panel (**f**) and (**g**). Panel **f** depicts the map resulting from standardization, whereas panel **g** shows additional normalisation

Our novel SPAM method involves three steps, namely standardisation, permutation, and cluster-enhancement, for the analysis of atherosclerotic plaque formation and tracer distribution in FRI images of murine aortic vessels. The overall workflow of the proposed method is depicted in Fig. 1. An anatomical overview of the analysed vessel region is presented in Fig. 2. First, we define regions of interest (ROIs) within the vessels for the subsequent analysis. Each region is transformed into a standardized map, i.e. the region of the vessel is unwarped (i.e. straightened) and its length and width rescaled to predefined values. Furthermore, the fluorescence intensity of these maps is normalized to improve comparability between different subjects. To compare two different groups of subjects, we then apply permutation testing in combination with a cluster-enhancement method. In the subsequent sections, we will describe each of the steps in detail. All images depict fluorescence intensity maps, which were visualized by application of a false colour palette.

### ROI definition

To acquire comparable ROIs across all subjects, we propose a semiautomatic approach. Although there are automated segmentation approaches for vessel structures (such as described in [15] and [16]), we propose a method relying on user-defined boundary points. This allows our method to be applied on a wide variety of different image modalities where multiple sets of parameters or even entirely different automated segmentation approaches would be necessary otherwise.

**Fig. 3:**
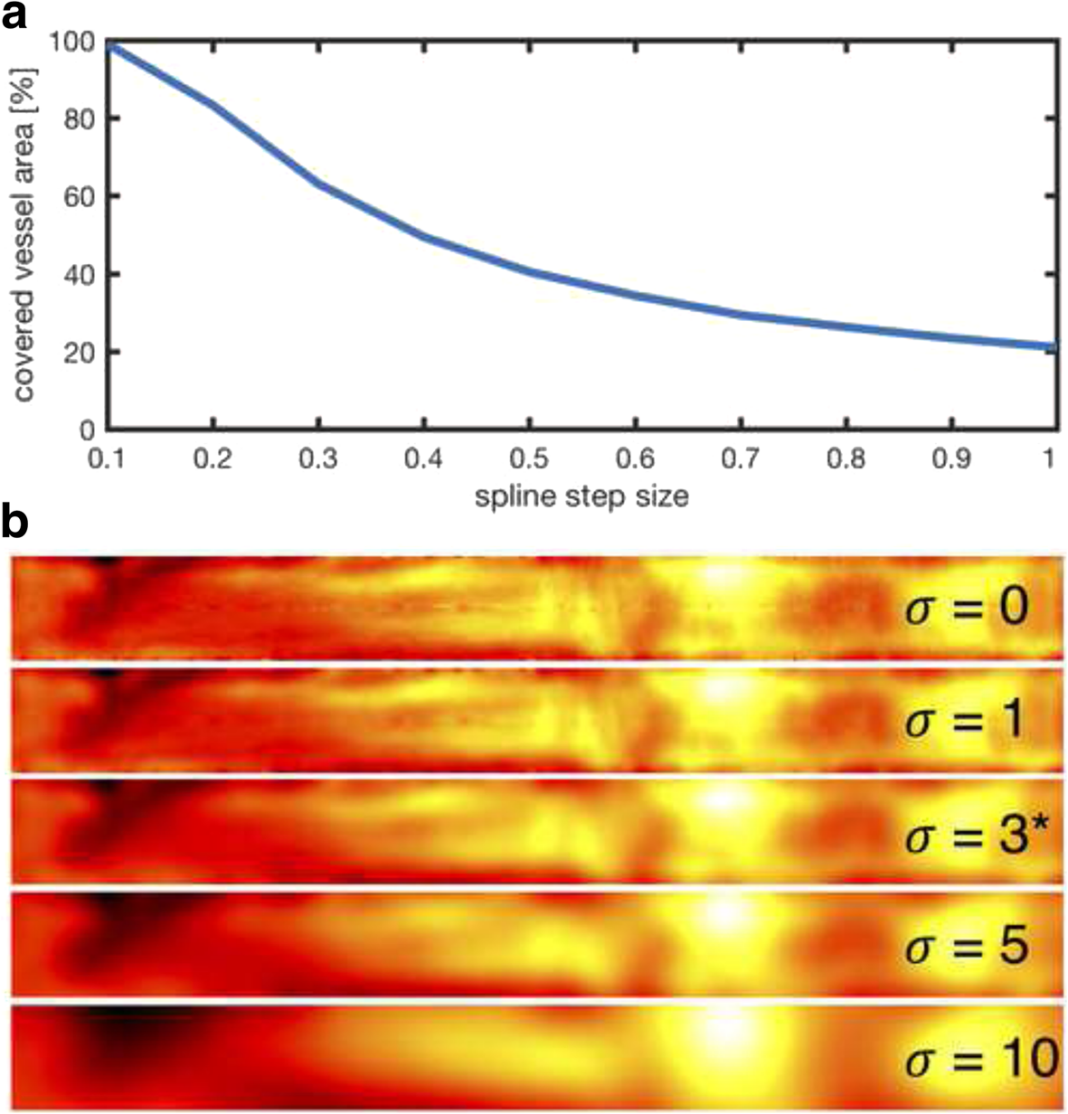
Panel **a** depicts the influence of the spline step size on the area of the vessel covered by the orthogonal line segments. The chosen step size of 0.1 (approximately 1.88 steps per pixel or a step size of 18 *μ*m) leads to a complete coverage of the underlying vessel area and is thus an optimal choice for unwarping the vessel, as no image information gets lost. Panel **b** highlights the influence of the Gaussian smoothing *σ* on the mean difference maps, which has been experimentally chosen as *σ*=3. For lower values of *σ*, noise and artifacts become visible in the mean difference maps, while for larger values, structures become less clear due to overly smoothing the maps

At first, several boundary points are marked manually along each of the two outer boundaries of the vessel to be analysed. Those points are then used to retrieve the vessel boundaries by performing a natural cubic spline curve interpolation [17]. An example of this process is depicted in Fig. 2 d. Each of the two outer aortic curves is evaluated at multiple positions, which results in a list of points along the curve constituting a piece-wise linear approximation of the curve, i.e. a polygonal chain. To obtain a sufficient number of points regarding the resolution of the image, we experimentally chose a step size of 0.1 along the spline. The step size is defined in terms of the spline curve length according to the centripetal scheme proposed by Lee [17] and corresponds to approximately 1.88 steps per pixel or a step size of 18 *μ*m. This results in nearly a thousand points per curve and ensures a complete overlap of the vessel area later on (see results section and Fig. 3 a).

In the next step, we compute orthogonal line segments from one vessel boundary curve to the other which are later used for unwarping the vessel. For each point along a polygonal chain A we compute its nearest neighbour on polygonal chain B and vice versa, resulting in a set of orthogonal line segments for each of the boundary chains. An example showing the resulting orthogonals for both aortic boundary curves is depicted in Fig. 2 a. Combining these two sets of line segments results in instances of crossing orthogonals (see Fig. 2 b). We then aim to find a subset of the line segments in the union of the two sets which best represents the area within the vessel so that in the unwarping process no information is lost, i.e. to prevent holes which might originate when choosing the line segments of the shorter boundary curve (see Fig. 2 a). This is achieved by choosing the line segments resulting from the locally longer curve (i.e., the curve at the outside of a bend) and switch at inflection points of the vessel if necessary. At such points (or straight vessel segments) two points are assigned to each other from both sides, i.e. they are mutually nearest neighbours. Figure 2 b depicts the line segments between those points using the red colour. The result of combining the sets of line segments using the aforementioned approach is a set of orthogonal line segments, which represents the entire area of the vessel (see Fig. 2 b). The line segments are used to generate a medial axis (centre line) of the vessel which is defined by a polygonal chain consisting of the centre point of each line segment, providing length information of the vessel or its anatomical sub parts.

Based on the set of orthogonal line segments, it is possible to choose orthogonals which define the boundaries of anatomical regions of interest. The first and last selected line segments thereby define the closure of the region, providing a mask for the ROI. To improve the comparability of the anatomical landmarks and reduce the difficulty of manually selecting corresponding sections in images of multiple subjects, we limit the selection to the non-crossing orthogonal line segments from the previous step. Figure 2 d shows an example of selected ROI sections using the dotted lines orthogonal to the vessel.

For analysing aortic vessels, the anatomical ROIs are defined as the vessel segments between the major branching points, as these may affect shear stress and are robust to identify across subjects. The major branching points are the aortic artery and the brachiocephalic artery, the left common carotid artery, and finally the left subclavian artery. Thereby homologues regions are formed that are suitable for comparison. Making use of the automatic generated centre line, it would even be possible to split up the sections into inner and outer curvature ROIs, taking into account the different flow patterns.

### Unwarping, standardization and normalisation

Using the orthogonal line segments and anatomical section definition outlined above, it is now possible to compute an intensity map along the vessel. This produces an unwarped version of the aortic vessel, which we refer to as map throughout the remainder of this article. To compute the map, we first create a temporary image by recording the image intensities along each of the orthogonal line segments computed in the previous step into a separate row of a new image. To account for the varying distance of the line segments along the vessel, we perform an additional correction step where the image is stretched according to the physical length of the vessel’s medial axis by resampling the rows using bi-cubic interpolation, resulting in the actual map. An example is depicted in Fig. 2 e.

To compare maps across subjects, each map must be standardized to a uniform length and width. Using the information of anatomical sections, the map’s sections are stretched to predefined lengths, again using bi-cubic interpolation. For our example data, we specified 5 sections (*A*_1_ to *A*_5_) which were standardized to the lengths *L*_1_=*L*_2_=*L*_3_=*L*_4_=50 pixels and *L*_5_=200 pixels to alleviate the problem of underrepresenting certain regions. Otherwise the aortic arch region would be relatively reduced in size due to strong bending. Moreover, each profile is scaled to a predefined width to compensate for lumen narrowing. For the whole map, a uniform width *W*=40 pixels was chosen. Those parameters can be adjusted by the user according to the specific application. To overcome spatial inaccuracies and signal noise, a Gaussian smoothing operation is applied to the maps. For the initial Gaussian smoothing, the parameter *σ*=3 was used. When computing the mean difference maps, for lower values of *σ*, noise and artifacts are visible in the images, while larger values lead to overly smoothed, i.e. blurry, images. An exemplary visualization of this parameter’s influence is shown in figure Fig. 3 b.

These standardized maps can be compared to each other, providing high anatomical accuracy. Furthermore, each map is normalized to the subject’s mean background signal intensity, e.g. the uninflamed left common carotid artery (see Fig. 2 c) to allow a comparison of the intensity maps. An example of the standardized and normalized map is depicted in Fig. 2 f).

Now that comparable maps have been computed, the following section will focus on the methodology of comparing those maps in a reliable way to identify statistically significant differences between groups of subjects.

### Permutation testing

A simple form of evaluating maps is to calculate an average map over a group of subjects. To compare two groups, a mean difference map, i.e. the difference image of the two average maps, can be computed. However, this method provides no statistical significance, as this information cannot be derived directly from the difference image. To overcome this problem, groups of standardised maps can be compared by means of pixel-wise permutation testing or cluster-wise permutation testing. This technique is already well-established in quantitative analysis of fMRI, e.g. [9, 10, 12]. The main idea of permutation testing is to build up an H0 distribution by randomly exchanging group labels of the measurements between the two test groups [10]. If the biggest difference between two groups is observed in the original assignment, this difference is likely not due to random variation [9]. Using the H0 distribution, one can check this assumption using a test statistic [18].

Permutation tests share the benefit that only few assumptions have to be met in order to be applicable [10]. However, one key assumption is that subjects are interchangeable between the two subject groups [10]. Unfortunately, there are circumstances when this assumption is not correct, such as in paired comparisons (e.g. comparing two tracers in the same individuals such that there is a pair of images for the same subject, each image in one of the two groups). Therefore, we apply two different permutation approaches, one for unpaired and one for paired comparisons.

#### Unpaired permutation

**Fig. 4:**
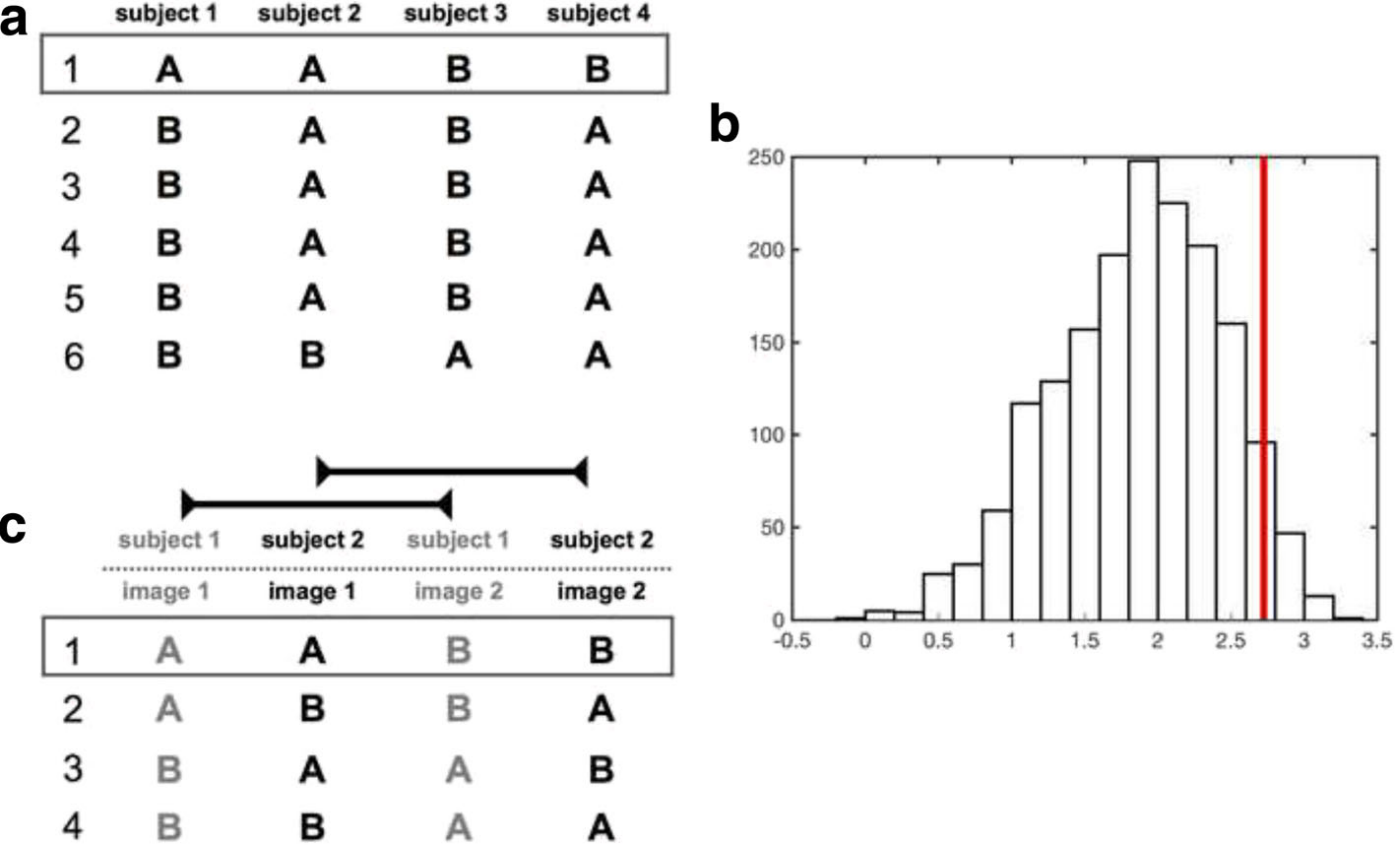
Panel **a** shows all possibilities for the label exchange of four subjects and the resulting combinations. The box highlights the true assignment of the subjects to test groups *A* and *B*. A sample maximum difference distribution is shown in panel (**b**). The desired 5% percentile is marked in *red*. It can be used to identify a threshold t which is then used for thresholding the normalized difference map. Panel **c** shows the label exchange of paired images which are acquired using the same subjects. Only images of the same subject are exchanged between groups. *Light grey colour* indicates corresponding images. The box highlights the true assignment of images to test groups

For permutation testing of two unpaired groups, the mean difference map is calculated for each of the groups. As mentioned previously, the goal is to eliminate insignificant differences, i.e. arbitrary differences that are also present when randomly exchanging the assignment of subjects to group A and B. This is done by sequentially computing permutations of the group labels which are assigned to the images (see Fig. 4 a) and calculating the mean difference map of the groups resulting from each permutation. For n subjects in total and *k*<*n* subjects in one of the groups, we obtain $\binom {n}{k}$ combinations in total [10]. Unfortunately, for large numbers of subjects, testing of all combinations is not feasible due to the large number of combinations [18]. For instance, even a moderate number of *n*=30 subjects with a group size of *k*=15 leads to over 155·10^6^ combinations in total. In such cases, it is possible to choose a random subset of permutations, which is sometimes referred to as Monte-Carlo permutation testing [10]. In general, selecting 1000 permutations is sufficient to obtain effective approximate permutation tests [10]. In our implementation, we restrict the maximum number of permutations to 5000 for instances where more combinations are possible.

The aforementioned mean difference maps lack the information on signal scatter. A high local difference suggesting a disparity might be misleading due to high signal scatter. Therefore, for each pixel, the standard deviation (SD) of its intensity is calculated over the mean difference maps of all permutations and used to normalize this pixel in each of the difference maps, i.e. the pixel’s intensity is divided by its SD [18]. The resulting maps are referred to as normalized difference maps for the remainder of this section. The maximum pixel value is calculated for each individual normalized difference map to derive a distribution of maximum values over the permutations. Thereby, permutation testing produces its own H0 test distribution. Since the distribution consists of the maximum difference values (regardless of the pixel location containing the maximum at a given permutation) it is taken account of the multiple comparisons problem. Using the acquired maximum difference distribution, the desired percentile (e.g. 5%, see Fig. 4 c) can be used to identify a threshold t which is then used for thresholding the normalized difference map computed using the original labelling of the images, i.e. the actual groups.

As the (normalized) difference maps contain signed values, we perform the same test using the minimum pixel value (i.e. maximum negative pixel value) on the normalized difference maps computed from the permutations, which allows us to identify significantly smaller pixels across groups analogue to its maximum counterpart. Some exemplary results of the whole procedure are included in the results section.

#### Paired permutation

A slightly different approach is used when group A and B are paired. In this situation, we apply the method proposed by Simpson et al. [19]. Only corresponding group members, i.e. images of the same subject, are exchanged between groups (see Fig. 4 b). If the true assignment of group A and B is significantly different for any pixel, swapping images of one subject results with high likelihood in a less extreme maximum or minimum difference value. The maximal amount of permutations is given by $2^{\frac {n}{2}}$. After building the test distribution, subsequent calculations are performed analogously to the paired permutation test. It should be noted that a comparison of two tracers is made possible using this method because of the aforementioned normalisation of each image to a predefined background region. Therefore, background normalized tracer images are compared to each other.

### Cluster-based enhancement

A limitation of the described permutation test is that it explicitly ignores cooperative effects between pixels. If a given pixel has a high value and its surrounding neighbours also show a comparable signal, it is more likely to be significant than if it is surrounded by pixels with low signal. As pixels should not be regarded as independent observations, it seems more suitable to perform a cluster-based statistic rather than a pixel-wise one. A cluster in this regard is a group of adjacent pixels with similar intensities.

**Fig. 5:**
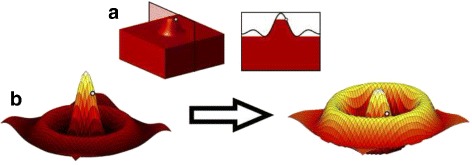
This figure highlights the function of cluster-enhancement on an artificial example signal. An exemplary point is marked in all of the panels. Panel **a** demonstrates that only locally connected regions directly under the given point contribute to the enhancement. Unconnected local maxima are ignored. Panel **b** shows a two-dimensional sample function expressed as a three-dimensional topological surface before and after cluster-enhancement is applied. The enhanced map is not only dependent on the original signal intensity, but also on the amount of surrounding pixels that show a similar signal intensity. This enhances local maxima in larger connected clusters of pixels

The *Threshold-Free Cluster Enhancement* (TFCE) approach proposed by Smith and Nichols [13] aims at solving the aforementioned problem by enhancing the image through means of adding local support by its neighbourhood to a pixel. Intuitively, the method interprets the two-dimensional image as a three-dimensional topological surface given by the pixel intensities and enhances local maxima of pixels in local clusters by incorporating the extent of the connected region below them (see Fig. 5 a). The cluster-enhanced value of a pixel *p* in the normalized difference map is computed by evaluating the following equation:

1 $$ enhanced~signal(p)= \int_{h_{0}}^{h_{p}} e(h)^{E} \cdot h^{H} dh,  $$

where *h*_0_ is the lowest intensity in the image, *h*_*p*_ is the original intensity of *p* in the normalized difference map, *e*(*h*) is the extent of the connected region under *p* at height *h*, and *E* as well as *H* are the additional parameters of the model. It should be noted that only locally connected regions directly under the pixel *p* at height *h* contribute to the enhancement so that unconnected local maxima are ignored (see Fig. 5 a). The TFCE-enhanced map is thus not only dependent on the original signal intensity, but on the amount of surrounding pixels that show a similar signal intensity. Therefore, TFCE supports pixels bundled to clusters to be more likely significant (see Fig. 5 b). For the parameters *E* and *H*, the standard values 0.5 and 2 have been used as defined by Smith and Nichols [13]. It should be noted that the TFCE method retains local maxima of the input image (although transforming their height) and that an iso-contour in the original image necessarily corresponds to an iso-contour in the TFCE-enhanced image, i.e. while TFCE is a non-linear transformation, it retains important basic features of the data [13]. In our implementation, a finite approach is used by thresholding the mean difference map at 500 levels between the image’s minimum and maximum. At each step, the size of a cluster containing the considered pixel is determined and multiplied with the current thresholding height *h*_*t*_.

We apply the cluster-enhancement in the permutation tests described above to each of the normalized difference maps, i.e. to the normalized difference map of each individual permutation (as well as the original labelling of the images). The aforementioned maximum and minimum distributions are thus already computed from the TFCE-enhanced values.

### Animal details and data acquisition

For our exemplary study, 13 ApoE deficient mice (Charles River Laboratories International, Inc., Wilmington, MA, USA) were fed a Western type diet (Altromin GmbH, Lage, Germany; 21% fat, 19.5*%* casein, 0.25*%* cholesterol) either for 12 (*n* = 6) or 17 (*n* = 7) weeks. At diet end, mice were injected intravenously with 2 nmol of the MMP photoprobe Cy5.5-AF443 [20]. Three hours past tracer administration, animals were euthanized (anaesthetized by 2% Isoflurane, 0.5l O_2_/min), perfused using a sodium chloride solution and dissected for the aortic artery explantation. To acquire the ex vivo fluorescence images, we employed an IVIS Spectrum Imaging System and the Living Image Software 4.0 (Caliper Life Sciences, Hopkinton, MA, USA).

## Results

One of the crucial steps of our proposed method is the calculation of a medial axis for unwarping the vessels to ultimatively obtain comparable regions of interest between different subjects. To validate the accuracy of our medial axis, we analyzed its position with respect to the two vessel boundary curves. For each pixel of the detected medial axis, we determined the pixels with the smallest distance on each of the two vessel boundaries. If the difference of those two distances is close to zero, the medial axis pixel is in the middle of the two boundaries, which is the optimal position. We evaluated 13 FRI data sets (see below) and obtained a mean difference of 0.3451 pixels with a standard deviation of 0.8636 pixels. Since both of those values are below one pixel, we can conclude that the medial axis is generally in a good position.

In addition to validating the medial axis in terms of its distance to the boundary curves, we performed an evaluation of the step size parameter for the boundary points of the spline curves with respect to the coverage of the vessel area by the orthogonals. The covered vessel area for different step sizes can be found in Fig. 3 a. The chosen step size of 0.1 leads to a complete coverage of the underlying vessel area and is therefore an optimal choice for unwarping the vessel, as the image information is completely retained. Increasing the step size decreases the covered vessel area considerably so that lower values would result in information loss in the unwarped maps.

**Fig. 6:**
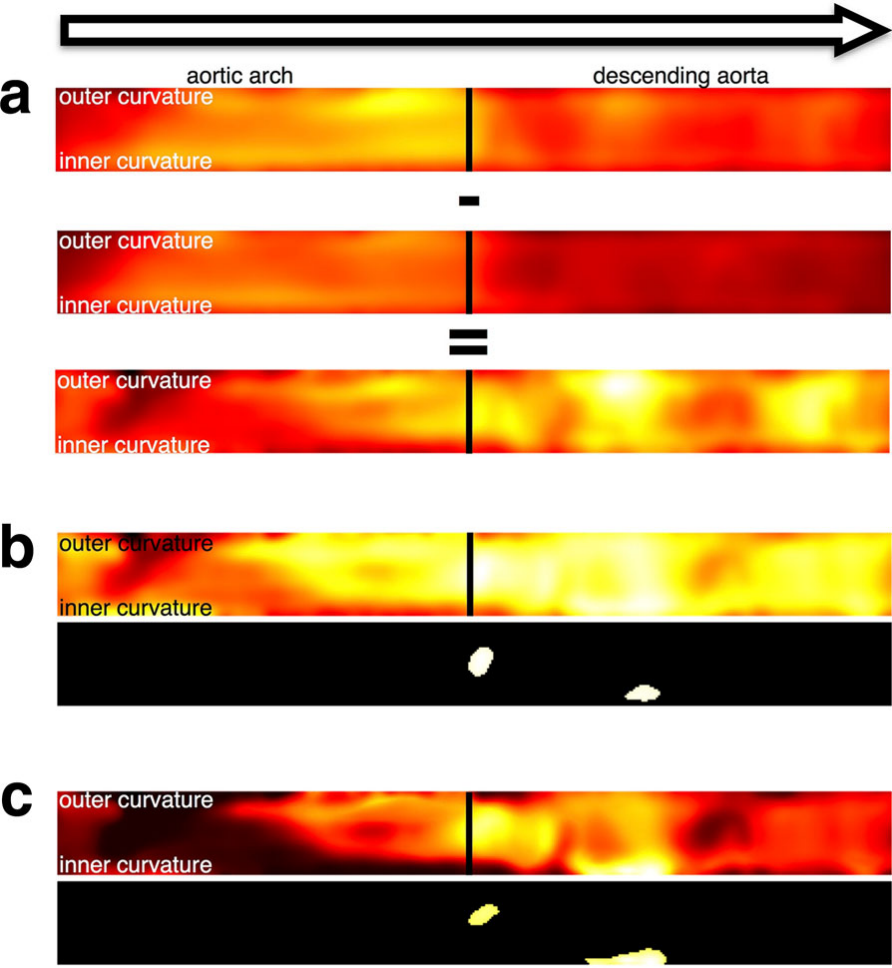
Panel **a** shows the average maps of two test groups as well as the mean difference map derived from those average images. The colour maps of the two average maps are scaled to the same maxima and minima. The *arrow* indicates blood flow of the unwarped and standardized aortic artery maps. Aortic arch, descending aorta and inner as well as outer curvature are marked. Panel **b** depicts the resulting normalized difference map after permutation testing as well as the thresholded difference map, where the threshold t has been derived from the maximum permutation distribution using the significance level *α* = 0.05. Panel **c** shows the same results with the addition of the cluster-enhancement

To evaluate our method as a whole, we used two groups of ApoE deficient mice (*n*=13 in total), which were fed with a high cholesterol diet for 12 or 17 weeks. Both the genotype and diet have been shown to promote the development of atherosclerosis [21]. The MMP binding Cy5.5-AF443 photoprobe was administered intravenously 3 h before image acquisition [20]. At the end of the diet animals were sacrificed, aortic vessels were dissected and ex vivo FRI images acquired. Aortic arteries were cut at the root proximally and at the diaphragm hiatus distally. Figure 6 a shows the average maps computed from the two groups as well as the mean difference map derived from those average images [22]. Figure 6 b shows the resulting normalized difference map after permutation testing as well as the thresholded difference map where the threshold t has been derived from the maximum and minimum distributions using the significance level *α*=0.05. Figure 6 c shows the same results with the addition of the TFCE. Note that the cluster of pixels significantly differing between group A and group B is difficult to identify in the normalized difference map of the first example, but clearly visible when applying the cluster-enhancement.

The greatest difference of Cy5.5-AF443 signal between the two diet groups (12 vs. 17 weeks) is located in the middle of the descending aortic artery. There is a significant hotspot of MMP activity adjacent to the inner curvature due to higher intensities of the 17 week’s average map (*p*<0.05). Additionally, the 17-week group shows more Cy5.5-AF443 signal compared to the 12-week group in the outer curvature of the aortic arch, but this difference is not significant (*p*>0.05).

## Discussion

In the current study we present a novel method, which we call SPAM, for analysing inflamed atherosclerotic aortic vessels in order to find significant patterns of tracer distribution in FRI images. In this respect, targeting MMPs by tracers seems a promising approach for imaging atherosclerotic plaque [23–26]. However, to evaluate the chosen Cy5.5-Af443 MMP tracer or any other tracer targeting atherosclerosis for identifying dangerous plaques, it is mandatory to use precise and standardized image analysis methods. Our method enables the definition of vascular ROIs in a highly reproducible and accurate manner using vessel branching points as landmarks. This allows us to compare results across subjects to study the development of atherosclerotic plaque and potential tracer uptake per vascular segment.

Alternatives to ROI-based image quantification have been published, like the combination of whole image sets to form standardized maps [5, 6]. To display and analyse vessel images in such a standardized manner, unwarping is a common procedure [27–29]. While we also apply unwarping, we use vascular branching points in our method to transform the image into a standardized map. This overcomes the topographical image variety consisting of positional and morphological contribution [7].

We demonstrated the vessel standardization using 13 FRI images of murine aortic vessels in total. Our strategy of average map generation and display provides a clear overview of the relevant image features. There are several other approaches to mask, refine and display vessel segmentation volumes [30–33]. However, these lack the possibility to test maps against each other.

The use of permutation testing provides a strong control over alpha errors in imaging settings [12]. There are other approaches to find significant changes between groups of maps, like Gaussian Random Field. However, these could lead to too liberal results due to violation of the Gaussian Random Field assumptions [11, 12]. Adjusting the *α*-value for every pixel using Bonferroni correction, on the other hand, may lead to a too conservative result [34]. Thus, the non-parametric permutation seems to be best suited.

Whole-map testing is especially useful as there is often no regional distribution hypothesis for atherosclerotic plaque. This could lead to excessive ROI definitions with an alpha error accumulation. In contrast, our approach enables the user to find significant patterns of plaque, which would not be visible without it. This is demonstrated by our data, indicating that the 17-week group bears more Cy5.5-AF443 signal activity in the descending part of the aorta than the 12-week group, which sets our approach apart from the general expectation that the aortic arch is a universal predisposed site of atherosclerotic plaque formation [35]. However, further studies are necessary to validate the correlation of FRI signal intensity with atherosclerosis.

The TFCE-enhanced map enables the viewer to find the hotspot more easily prior to the thresholding operation. Furthermore, the distal significant cluster is bigger compared to the non-TFCE result. This distal cluster is located at the prolongation of the inner curvature which is a well described predilection side for atherosclerosis [35].

We expect that our method will provide a useful tool for the field of molecular imaging of atherosclerosis. Although vessel sites prone to atherosclerotic plaque formation have been described, very little is known about the underlying biophysical parameters. This results in a lack of a priori knowledge that would be needed to define meaningful ROIs for specific vessel parts. Our standardization and testing method does not need this a priori information and is therefore able to study atherosclerotic vessels in an unbiased manner. Moreover, it helps to gain a deeper understanding of the underlying biophysical parameters and thereby leads to valuable a priori knowledge for future molecular imaging approaches.

The presented method is equally applicable to image information obtained in vivo. However, FRI images are not easily acquired in vivo due to limitations of tissue preparation and light scattering caused by adjacent anatomical structures. Yet we do believe that suitable in vivo data could become available soon, e.g. through highly refined intravital multiphoton microscopy approaches. To fine tune and validate our methodology, ex vivo data seems to be the most robust and appropriate choice.

In future work, we will extend our method to the analysis of three-dimensional datasets (e.g. acquired by PET). For doing so, the specification of orthogonal slices through the aortic vessel must be modified to take care of three-dimensional spill-in artefacts. The remaining parts of SPAM can easily be adjusted to volume analysis [32, 36]. Furthermore, we want to include more automated segmentation methods in the ROI definition step of our workflow for specific image modalities.

## Conclusion

We propose a novel method for analysing molecular images with regard to atherosclerosis plaque formation which is based on a semi-automatic standardisation approach in combination with permutation testing and a cluster enhancement method. Although permutation tests are already well-established in other areas, to the best of our knowledge such methods have not been applied to molecular image analysis so far. We tested our method using FRI images of murine aortic vessels in order to find recurring patterns of atherosclerotic plaque across multiple subjects. In addition, we have shown that cluster-enhanced permutation testing is a powerful approach for finding significant differences of tracer distributions in atherosclerotic inflamed vessels.

## Abbreviations

AA: Aortic arch
APO E: Apolipoprotein E
BA: Brachycephalic artery
CCA: Common carotid artery
DA: Descending aorta
EEG: Electroencephalography
fMRI: Functional magnetic resonance imaging
FRI: Fluorescence reflectance imaging
MMP: Matrix metalloproteinase
PET: Positron emission tomography
ROI: Region of interest
SA: Subclavian artery
SPAM: Statistical permutation- based artery mapping
SPECT: Single photon emission computed tomography
SPM: Statistical parametric mapping
TFCE: Threshold-free cluster enhancement
